# Period3 modulates the NAD^+^-SIRT3 axis to alleviate depression-like behaviour by enhancing NAMPT activity in mice

**DOI:** 10.1016/j.jare.2025.01.043

**Published:** 2025-02-01

**Authors:** Xiaoxian Xie, Haoshen Xu, Ruonan Shu, Shulin Du, Haidan Fan, Mengya Zhang, Lei Sun, Jiafeng Zhou, Liangliang Wang, Zezhi Li, Daniel C. Anthony

**Affiliations:** a Shanghai Mental Health Center, Shanghai Jiao Tong University, School of Medicine, Shanghai 201108, China; b Department of Pharmacology, University of Oxford, Mansfield Road OX1 3QT, Oxford, UK; c Shanghai Key Laboratory of Psychotic Disorders, Brain Health Institute, Shanghai Mental Health Center, Shanghai 201108, China; d College of Biotechnology and Bioengineering, Zhejiang University of Technology, Hangzhou 310032, China; e Department of Nutritional and Metabolic Psychiatry, Affliated Brain Hospital, Guangzhou Medical University, Guangzhou, China; f Key Laboratory of Neurogenetics and Channelopathies of Guangdong Province and the Ministry of Education of China, Guangzhou Medical University, Guangzhou, China; g College of Animal Science, South China Agricultural University, Guangzhou 510640, China; h College of Ecology, Lishui University, Lishui 323000, China

**Keywords:** Depression-like behavior, Per3, NAD^+^, Mitochondrial complex, Energy metabolism, SIRT3

## Abstract

**Introduction:**

PERIOD (PER)3 deficiency is associated with depression-like behaviors, but the underlying mechanisms remain unclear.

**Objectives:**

This study aims to elucidate the role and mechanism of PER3 in regulating depression-like behaviors in mice.

**Methods:**

Depression-like behaviors were assessed using the sucrose preference test, tail suspension test, and forced swimming test. Metabolomic analysis was conducted on hippocampal tissues from Per3 knockout mice using chromatography-mass spectrometry. The regulatory role of PER3 on the expression of nicotinamide phosphoribosyltransferase (Nampt) was investigated through co-immunoprecipitation and chromatin immunoprecipitation assays.

**Results:**

Metabolomic analysis revealed that Per3 deficiency disrupts mitochondrial function, as evidenced by reduced activities of key tricarboxylic acidcycle enzymes (succinate dehydrogenase, citrate synthase, and α-ketoglutarate dehydrogenase), diminished expression of mitochondrial respiratory chain complexes I-V, and decreased nicotinamide adenine dinucleotide (NAD)^+^ levels in Per3 knockout mice. Supplementation with the NAD^+^ precursor nicotinamide rescued mitochondrial function and alleviated depression-like behaviors in Per3 knockout mice. Similar effects were observed with intraperitoneal administration of the NAMPT activator P7C3-A20, while these effects were abolished by the NAMPT inhibitor FK866. Mechanistically, PER3 was found to regulate Nampt expression by binding to E-box elements within its intronic regions in conjunction with BMAL1. This interaction enhanced NAD^+^ production, activating SIRT3 to mitigate mitochondrial dysfunction in Per3 knockout mice.

**Conclusions:**

These findings uncover a novel mechanism by which PER3 ameliorates depressive behaviors through the regulation of NAMPT-controlled NAD^+^ levels and mitochondrial function, underscoring the critical role of PER3 in depression-related pathophysiology.

## Highlights

• Per3 deficiency reduces NAD^+^ levels in the brain and induces depression-like behaviors in mice.
• Enhancing NAD^+^ levels through NAM supplementation or NAMPT activation alleviates depression-like behaviors in Per3 knockout mice.
• NAD^+^-mediated modulation of SIRT3 activity links mitochondrial dysfunction to Per3-induced depression.
• PER3 regulates Nampt expression by binding to its promoter region, controlling NAD^+^ levels and mitochondrial function.

## Graphical abstract

**Figure:**
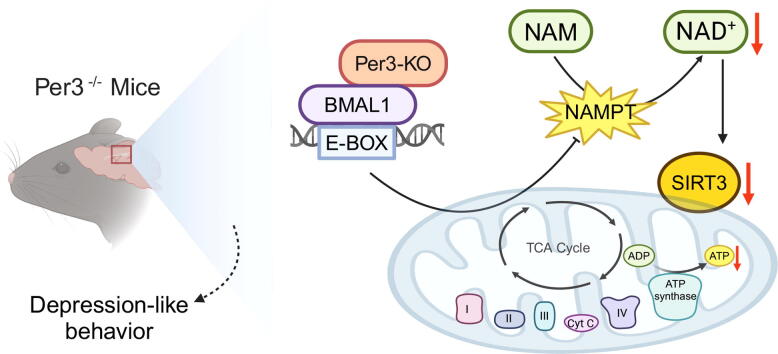

## Introduction

Three period (Per) genes, Per1, Per2, and Per3, have been identified as the key components of the circadian clock, playing essential roles in its regulation [1]. However, a previous study reported that Per3 knockout mice can maintain a diurnal rhythm as long as one of Per1, Per2, Cryptochrome (Cry) 1, or Cry2 genes are present [2], suggesting Per3 is not an essential gene for maintenance of circadian rhythms [3]. The variants of Per3 have been extensively studied and are associated with certain distinct phenotypes, including diurnal preference [4], sleep-wake disturbances [5], [6], altered cognitive performance [7]. Strikingly, polymorphisms of Per3 were also shown to be correlated with mood health [8] and psychiatric disorders [9]. Furthermore, Per3 has been reported to influence mental health, and Per3 knockout mice have been shown to exhibit depression-like behavior. [10]. These studies suggest a potential role of Per3 in the occurrence of depressive behavior through mechanisms that are likely to be separate and distinct from its role in the control of circadian rhythms.

Studies have identified a significant link between circadian rhythms and mitochondrial function. The circadian clock regulates mitochondrial protein abundance, with many proteins peaking during the early light phase [11]. PER1 and PER2 proteins, for example, play a crucial role in optimizing mitochondrial metabolism to daily changes in energy demand [12]. Recent work has indicated an increased risk of severe psychiatric disorders, including major depression, in patients suffering from mitochondrial dysfunction-related pathologies [13]. For example, research has uncovered a significant link between Mitofusin-2 (MFN2) in the nucleus accumbens (NAc) and the regulation of anxiety and depression-like behaviors. Highly anxious animals exhibit reduced MFN2 expression in the NAc, along with alterations in mitochondrial function and neuronal structure [14]. A significant association between mitochondrial DNA (mtDNA) levels in the blood or postmortem brain samples and depression has been reported [15], and mtDNA expression and content in occipital cortex samples are decreased in major depressive disorder patients when compared with control subjects [16]. It is also of note that mice with mitochondrial DNA polymerase knockout displayed neuronal mitochondrial dysfunction that was coupled with depressive-like symptoms [17]. However, the causal relationship between depression and mitochondrial function remains unclear.

Here, therefore, we sought to examine the relationship between Per3 and mitochondrial energy metabolism in regulating depressive phenotype. Our metabolomic analysis suggests that Per3 deficiency is associated with disruption of mitochondrial function, as we revealed a reduction in the activities of key enzymes in the  tricarboxylic acid (TCA)  cycle and activities of complexes I-V, as well as a decreased level of nicotinamide adenine dinucleotide (NAD)^+^ in the Per3 knockout mice. Next, we showed that supplementation of nicotinamide (NAM) can rescue the impaired phenotype, as well as the nicotinamide phosphoribosyltransferase (NAMPT) activator P7C3-A20. The effects were abolished by the injection of NAMPT inhibitor FK866. Furthermore, we identified that PER3 can regulate the expression of Nampt by binding to its intron E-boxes. Mechanistically, BMAL1 and PER3 bind the E-box1 element to regulate the expression of Nampt. Taken together, we suggest Per3 ameliorates depressive disorder through modulating the NAMPT-controlled NAD^+^-SIRT3 pathway to regulate the mitochondrial energy metabolism in the hippocampus of mice.

## Materials and methods

### Mice

Male and female heterozygous mice (Per3^+/−^) were obtained from the Nanjing Model Animal Center. 8-week-old male and female heterozygous mice were co-housed in the same cage for breeding, and offspring mice were obtained. Genotyping was performed by collecting clipped nails from the offspring mice to identify and select homozygous knockout (Per3^−/−^) mice. These homozygous knockout mice were further bred by pairing eight-week-old male and female homozygous Per3^−/−^ mice to expand the breeding programme and obtain male Per3^−/−^ mice for subsequent experiments.

Wild-type (WT) male C57BL/6J mice were purchased from the China Experimental Animal Resource Center (Shanghai). All experimental animals were kept in a controlled environment simulating natural day-night cycles, with the temperature maintained at 22–25 °C and a light–dark cycle of 12 h light and 12 h darkness (12L:12D, lights on at 8 AM, lights off at 8 PM) with free access to food and water. All experimental mice were acclimatized to these constant conditions for one week before the commencement of the experiments.

### Drug preparation and administration

NAM and FK866 were dissolved in DMSO and subsequently diluted in physiological saline for intraperitoneal injection into the Per3^−/−^ mice. NAM was administered once daily at a dose of 300 mg/kg, while FK866 was injected twice daily at a dose of 10 mg/kg; the injections were performed in the morning and evening. WT mice and Per3^−/−^ mice received intraperitoneal injections of physiological saline containing 10 % DMSO once daily as controls.

P7C3-A20 was dissolved in corn oil containing 10 % DMSO and administered to Per3^−/−^ mice via an intraperitoneal injection once daily at a dose of 28 mg/kg. The control groups, comprising WT mice and Per3^−/−^ mice, received intraperitoneal injections of 0.2 mL corn oil containing 10 % DMSO daily.

The treatment duration for all compounds, including NAM, FK866, and P7C3-A20, was 2 weeks.

### Behavioral assays

#### Sucrose preference test

The sucrose preference test was carried out as previously described [18]. For the first 24 h, animals were habituated with two bottles of 2 % sucrose solution. After fasting for 24 h, one bottle of the 2 % sucrose solution and one bottle of water was provided to each cage, and the position of the two bottles were switched after 30 min, then the consumption of sucrose solution and water were measured after 1 h. Sucrose preference = (consumed sucrose)/(consumed sucrose + consumed water) × 100 %. The test is carried out in a double-blind method.

#### Tail suspension test (TST)

Mouse was suspended by their tail with tape on the ceiling in its own three-walled rectangular compartment (30 cm height × 15 cm width × 15 cm depth). The mouse was suspended in the middle of this compartment and there was sufficient space for the mouse to struggle. The behavior of each mouse was videotaped and the immobile time (cessation of limb movements) during the last 4 min of a 6 min session was determined.

#### Forced swimming test (FST)

All mice were placed individually into a transparent circular water tank with a diameter of 0.5 m and a height of 0.8 m, where the water depth was 0.6 m and the water temperature was maintained at 25 ± 1 °C. The mice were deemed to be immobile when they floated without struggling. Each mouse was tested for 6 min, and the mouse was scored for immobility during the last 5 min; the test was performed in a double-blind fashion.

### Metabolomics analysis

GC/MS-based hippocampus metabolomics assay was carried out as in our previous study [19] with minor modifications. Briefly, the hippocampus from WT and Per3 knockout mice were selected for metabolomics analysis with four biological replicates in each group. The samples (∼28 mg) were extracted with 0.24 mL extraction liquid, and then were treated following standard procedures, and finally were analysed with an Agilent 7890 gas chromatograph system equipped with a Pegasus HT time-of-flight mass spectrometer (Agilent 7890 A, Agilent Technologies, USA). The differential metabolites were sorted based on a statistically significant threshold of changed impacts on VIP values, which were obtained from the OPLS-DA model, and *p* values from a two-tailed Student’s *t*-test on the normalized peak areas, where the metabolites with VIP values > 1.0 and *p* values < 0.05 were included.

### Cell isolation, culture and inhibition or activation of SIRT3 enzyme in cells

The hippocampus primary cells were isolated from WT and Per3 knockout mice as a previous study [20]. Then, cells were cultured in 6-well plates containing 5 mL of Dulbecco modified Eagle medium (DMEM; Gibco) supplemented with 10 % fetal calf serum, and the medium was changed every 3 days. Once cells reached 85–90 % confluency, they were passaged for sub-culturing.

To inhibit or activate the SIRT3 enzyme, cells were incubated in a 50 μM of SIRT3-selective inhibitor 3-TYP (MedChemExpress, Shanghai, China), or were incubated in 10 μM of SIRT3-specific activator honokiol (HKL) (Selleck Chemicals, USA) solution prepared in incubation buffer.

### Western blot

Western blot analysis was carried out as previously described [21]. The hippocampus was homogenized in 150 μL of 1 × RIPA (EMD Millipore Corp., Billerica MA, USA) with enzyme inhibitor, and allowed to stand for 10 min. The supernatant was isolated using centrifugation at 12000 rpm for 10 min, 4 °C. The total protein concentration was determined using a BCA protein assay kit (Thermo Fisher Scientific，USA). Aliquots (∼20 μg of total protein) were separated on SDS PAGE Gels and transferred to a polyvinylidene fluoride membrane. Then, the target protein area was selected and washed with 1 × TBST once, blocked for 1 h at room temperature with 3 % bovine serum albumin containing 0.1 % Tween-20 in buffer salt water. Then, it was incubated with primary antibodies against Total OXPHOS Rodent (1:250; Abcam, Burlingame, CA, USA), β-actin (1:5000; Cell Signaling Technology, Berkeley, CA), overnight at 4 °C. The blots were then washed three times in TBST for 5 min each and then incubated with secondary antibody for 1 h in TBST. After washing, immunoreactivity was tested using chemiluminescence with ECL reagent (Haokebio, China), and quantification of the band was performed with Image J software and normalized to β-actin levels within the same lane of the PAGE gel.

### Quantitative real-time polymerase chain reaction (qRT-PCR)

Total RNA was extracted from hippocampus using Biozol Reagent (Biomiga, China). Subsequently, the reverse transcription kit (Vazyme, China) was used for reverse transcription to obtain cDNA following the manufacturer’s protocol. PCR reaction was carried out by Roche Lightcycler 480. Quantification was examined using the comparative threshold cycle (Ct) measurement with SYBR green fluorescence signal (Applied Biosystems, Thermo Fisher Scientific). Expression levels of the selected genes (specific primers are shown in Table S1) were normalized to the level of β-actin gene. β-Actin was serviced as an internal control.

### Measurement of enzyme activities and enzyme-linked immunosorbent assay (ELISA) assay

Hippocampal tissue was homogenised at low temperature. SIRT3, SIRT4, SIRT5, NAMPT, succinate dehydrogenase (SDH), Citrate synthase, α-ketoglutarate dehydrogenase, ATP synthase activity was determined using kits (Abcam, Cambridge, MA, USA). The activity of SIRT3 (Abcam) and NAMPT (Abcam) were measured using an Enzyme Activity Assay Kit, and the ATP synthase was analyzed using an Enzyme Activity Microplate Assay Kit (Abcam) according to the manufacturer’s protocol. The kits for complex I-V (ATP synthase) were purchased from Sigma-Aldrich, and the kits for NAD^+^ were purchased from JingMei (Jiangsu, China), and the ATP level was detected using kits purchased from the Nanjing Institute of Bioengineering. The levels of complex I-V, NAD^+^ and ATP were detected using ELISA. All experiments were performed strictly according to the instructions of manufacturer.

### Transient transfections and reporter assays

The reporter plasmids were co-transfected into HEK293T cells along with expression plasmids containing the full-length cDNA of Per3 and Bmal1, or control plasmids, using Lipofectamine 3000 (Invitrogen) in 24-well plates. Luciferase assays were subsequently performed using the Luciferase Assay Kits (Promega, Madison, WI, USA), following the manufacturer's protocol.

### Co-immunoprecipitation (Co-IP)

Co-IP was performed using a Co-IP kit, following the manufacturer's instructions. Briefly, a portion of the lysate was set aside as the input, while the remaining lysate was incubated overnight at 4 °C with anti-BMAL1 (Abcam), anti-PER3 (Abcam), or IgG antibodies for immunoprecipitation. Magnetic beads were then added to bind the antibody-protein complexes. The samples were denatured by heating at 100 °C for 10 min, followed by elution. The resulting supernatant was collected for Western blot analysis.

### Chromatin immunoprecipitation (ChIP) assay

ChIP assay was carried out using a simple ChIP Plus Enzymatic Chromatin IP Kit (Cell Signaling Technology, Beverly, MA, USA), following the manufacturer’s instructions. Briefly,  primary mouse embryonic fibroblasts were cross-linked in 1 % formaldehyde for 10 min at room temperature. After the cross-linking, cells were washed with pre-chilled PBS, followed by digestion with micrococcal nuclease for 20 min. The supernatant was collected, and immunoprecipitation was carried out using anti-PER3 (Abcam, Cambridge, UK) and anti-BMAL1 (Abcam, Cambridge, UK) antibodies or normal IgG. Samples were incubated with protein A/G agarose/sepharose that precipitated endogenous DNA-protein complexes. Then, the cross-linking was reversed. Finally, proteinase K was added to the reaction mix to recover DNA fragments. qPCR was performed to quantify the amount of precipitated DNA bound to the target protein. The specific primers are listed as follows: E-box1 forward, 5ʹ- AACTCGAGTAGGTAACAGCCCCTGGCT −3ʹ; reverse, 5ʹ- AAAAGCTTTTAAAGCAATGTGCCATTTTACTC −3ʹ), and control site (forward, 5ʹ- AACTCGAGATACTTCAGATACTTTAAGTCATTCTGT −3ʹ; reverse, 5ʹ- AAAAGCTTACAGATGTGTTTGCAAACTACAGA −3ʹ).

IP was carried out using the nuclear complex IP kit, as per the manufacturer’s instructions. The SDS-PAGE fractionation and immunoblot assay were performed using anti-BMAL1 (Abcam) or PER3 (Abcam) antibodies.

### Ethics statement

All experiments involving animals were conducted according to the ethical policies and procedures were approved by the Animal Care and Use Committee at Zhejiang University of Technology and abided by the Guide for the Care and Use of Laboratory Animals of the National Institutes of Health (Approval no. 2020041039).

### Statistical analysis

GraphPad Prism 8.0 was used for data analysis. Data normality was determined by the D’Agostino-Pearson normality test. Results were expressed as mean ± SEM. Difference between two groups was analyzed by unpaired Student’s *t* test. *p* value < 0.05 for two-sided tests was considered significant.

## Results

### Per3 knockout alters metabolomics analysis in the hippocampus of mice

**Fig. 1:**
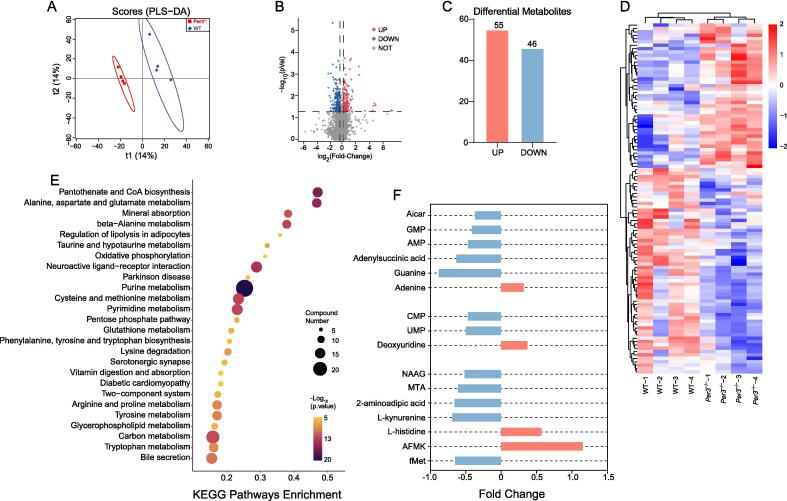
**Metabolomics analysis in the hippocampus of Per3^−/−^ mice.** (A) The PLS-DA model of GC–MS data derived from the hippocampus of WT group (blue dots) and Per3^−/−^ group (red dots). (B) The volcano plot of metabolites in the hippocampus between WT and Per3^−/−^ group. The red and blue dots expressed the up and down metabolites, and the grey indicate unchanged metabolites. (C, D) The quantity of altered metabolites and heat map of changed metabolites between WT and Per3^−/−^ group. (E, F) The analysis of significantly changed metabolites, and KEGG pathways between WT and Per3^−/−^ group. n = 4 per group.

As reported in a previous study [10], Per3 knockout mice showed depression-like behavior, which was consistent with our study; our animals exhibited reduced sucrose preference, increased immobility time in the FST and TST (Fig. S1). Then, we performed profiled the metabolome using GC–MS to detect the difference between Per3 knockout and WT mice. The data revealed clear separation between samples from Per3 knockout and WT mice following analysis with a supervised partial least squares-discriminant analysis (PLS-DA) model (Fig. 1A). A total of 101 metabolites were significantly altered in Per3 knockout mice, with 55 metabolites showing increased levels and 46 metabolites showing decreased levels (Fig. 1B, C), as visualized in the heatmap (Fig. 1D). Key metabolites, including adenine, deoxyuridine, L-kynurenine, guanine, and arylformamidase (AFMK), were closely associated with amino acid metabolism, mitochondrial energy metabolism, and oxidative stress (Fig. 1E). Additionally, pathways related to mitochondrial function were identified, such as pantothenate and CoA biosynthesis, alanine, aspartate, and glutamate metabolism, mineral absorption, β-alanine metabolism, and oxidative phosphorylation (Fig. 1F). Together, these findings suggest that Per3 knockout induces depression-like behavior, potentially through disruptions in mitochondrial energy metabolism in the hippocampus of mice.

### Per3 knockout impairs mitochondrial function by potentially reducing NAD^+^ levels

Based on the metabolomics data suggesting that Per3 modulates mitochondrial energy metabolism, we investigated its impact on mitochondrial function in the hippocampus of mice. Key indices of mitochondrial bioenergetics were measured using methods such as qRT-PCR, Western blot, and ELISA to evaluate mitochondrial functional markers.

**Fig. 2:**
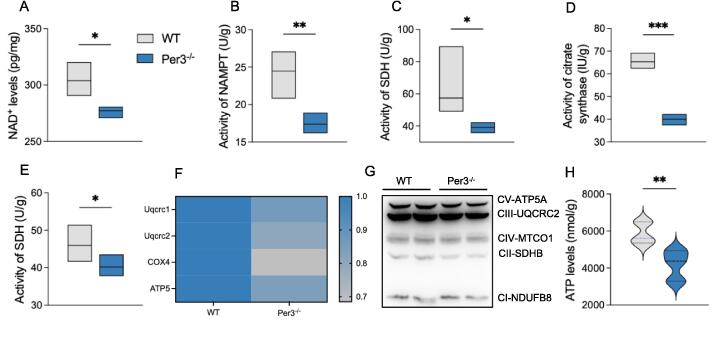
**Effects of Per3 knockout on behaviors and mitochondrial bioenergetics in the hippocampus of mice.** (A) NAD^+^ levels, (B-E) Activity of NAMPT, SDH, citrate synthase, α-ketoglutarate dehydrogenase, (F) mRNA levels of Uqcrc1, Uqcrc2, COX4 and ATP5, (G) Western blot analysis of oxidative phosphorylation system complexes (n = 3 per group), (H) ATP levels. Values represent the mean ± SEM. n = 8 in all groups in unspecified ones. *p < 0.05, **p < 0.002, ***p < 0.0002 vs. control groups.

Compared to WT mice, Per3 knockout mice exhibited a significant reduction in NAD^+^ levels and NAMPT activity in the hippocampus (Fig. 2A, B). Activities of key enzymes in the TCA cycle, including SDH, citrate synthase, and α-ketoglutarate dehydrogenase, were also markedly decreased (Fig. 2C-E). Moreover, the mRNA and protein expression of respiratory chain complexes I-V were significantly reduced in the hippocampus of Per3 knockout mice (Fig. 2F-G).

These changes were accompanied by a decrease in ATP levels (Fig. 2H) and evidence of increased oxidative damage. Markers of oxidative stress included elevated reactive oxygen species production, reduced glutathione content, and decreased mRNA expression of antioxidant enzymes such as SOD1, SOD2, GSR, and GPx (Fig. S2). Together, these findings indicate that Per3 knockout disrupts mitochondrial function in the hippocampus, potentially through a reduction in NAD^+^ levels.

### Enhancement of NAD^+^ level rescues the depression-like behavior and mitochondrial function in Per3 knockout mice

**Fig. 3:**
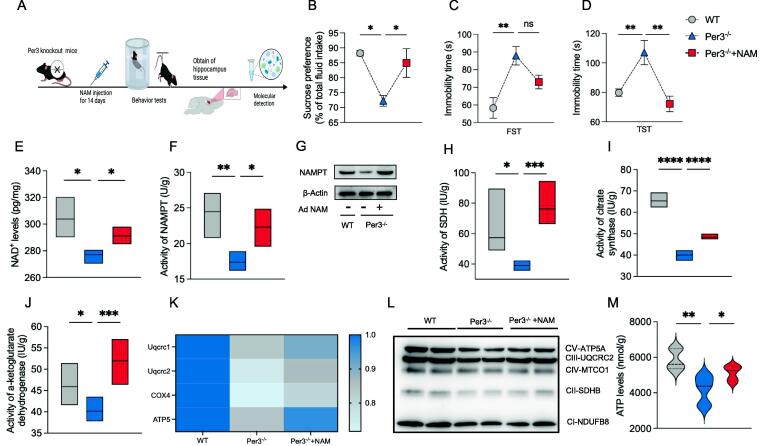
**Effects of NAM supplementation on depression-like behavior and mitochondrial function in the hippocampus of Per3^−/−^ mice.** (A) Experimental procedure diagram on Per3^−/−^ mice after NAM administration. (B) Sucrose preference test, (C, D) Immobility time in the TST, and TST. (E) NAD^+^ levels, (F) Activity of NAMPT, (G) Protein expression of NAMPT, (H) Activity of SDH, (I) Activity of citrate synthase, (J) Activity of a-ketoglutarate, (K) mRNA levels of Uqcrc1, Uqcrc2, COX4 and ATP5, (L) Western blot analysis of oxidative phosphorylation system complexes in hippocampus of mice (n = 3 per group), (M) ATP levels in the hippocampus of mice. Values represent the mean ± SEM. n = 8 in all groups in unspecified ones. *p < 0.05, **p < 0.002, ***p < 0.0002 vs. control groups.

Given the significant reduction in NAD^+^ levels observed in Per3 knockout mice (Fig. 2A), we sought to determine whether restoring NAD^+^ levels could rescue the mitochondrial dysfunction and reduced ATP production caused by Per3 deficiency. In mammalian cells, NAM serves as the predominant NAD^+^ precursor [22], Therefore, we administered NAM to Per3 knockout mice to increase NAD^+^ levels (Fig. 3A).

Behavioral assays showed that NAM administration alleviated depressive-like behavior in Per3 knockout mice, as evidenced by increased sucrose consumption (Fig. 3B) and reduced immobility in the TST (Fig. 3C, D).

We next investigated the effect of NAM on mitochondrial function in the hippocampus of Per3 knockout mice. NAM administration significantly restored NAD^+^ levels in the hippocampus (Fig. 3E), indicating activation of the NAD^+^ salvage synthesis pathway [22], Similarly, NAM treatment rescued both the activity and protein expression of NAMPT (Fig. 3F, G).

Additionally, NAM administration enhanced the activities of three key enzymes in the TCA cycle (Fig. 3H-J) and restored the mRNA and protein expression of respiratory chain complexes I-V in the hippocampus of Per3 knockout mice (Fig. 3K, L). Importantly, ATP levels were also significantly restored (Fig. 3M).

These findings demonstrate that NAM administration can effectively rescue mitochondrial bioenergetics in the hippocampus of Per3 knockout mice.

### FK866 impairs the effect of NAM on the depression-like behavior and mitochondrial function in Per3 knockout mice

**Fig. 4:**
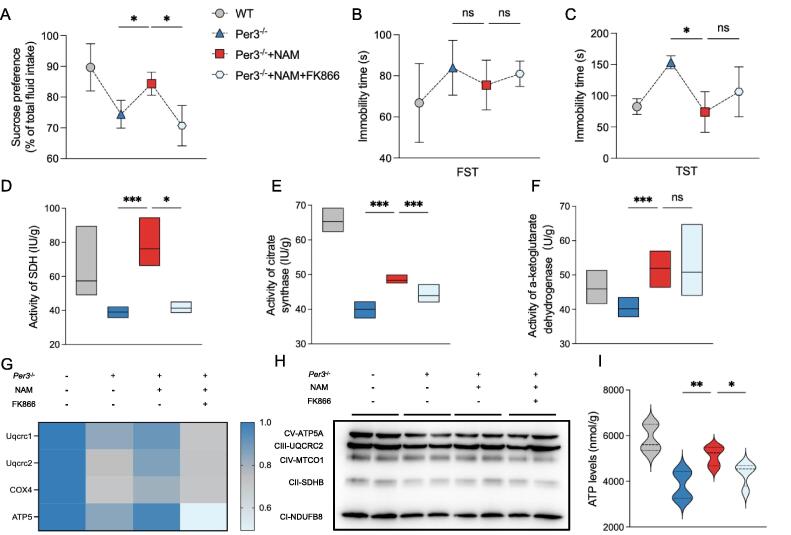
**Effects of NAMPT inhibitor FK866 on depression-like behavior and mitochondrial function in NAM-supplemented Per3^−/−^ mice.** (A) Sucrose consumption in the sucrose preference test, (B, C) Immobility time in the TST, and in the FST. (D-F) Activities of SDH, citrate synthase, and a-ketoglutarate, (G) mRNA levels of Uqcrc1, Uqcrc2, COX4 and ATP5, (H) Western blot analysis of oxidative phosphorylation system complexes (n = 3 per group), (I) ATP levels in the hippocampus of mice. Values represent the mean ± SEM. n = 8 in all groups in unspecified ones. *p < 0.05, **p < 0.002, ***p < 0.0002 vs. control groups.

NAMPT is the putative rate-limiting step in the NAD^+^ salvage pathway, and FK866 is the specific inhibitor of NAMPT [23]. Our results showed that FK866 treatment partly counteracted the effect of NAM on depression-like behavior by decreasing the sucrose consumption, though no effect was observed in FST and TST tests (Fig. 4A-C). In addition, FK866 treatment counteracted the effect of NAM on mitochondrial function in the hippocampus of Per3 knockout mice, including the activities of SDH and citrate synthase, mRNA and protein expression of partial complexes I-V (Fig. 4D-H). Finally, the significant reduction of ATP was observed after FK866 treatment in NAM administrated Per3 knockout mice (Fig. 4I).

### P7C3-A20 rescues the depression-like behavior and impairment of mitochondrial function induced by Per3 knockout

**Fig. 5:**
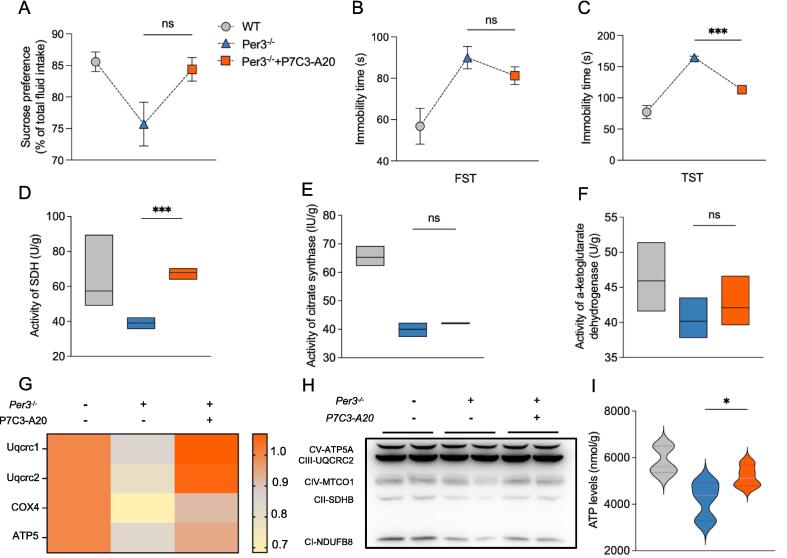
**Effects of P7C3-A20 on depression-like behavior and mitochondrial function in Per3^−/−^ mice.** (A) Sucrose consumption in the sucrose preference test, (B, C) Immobility time in the TST and the FST, (D-F) Activities of SDH,citrate synthase, and α-ketoglutarate, (G) mRNA levels of Uqcrc1, Uqcrc2, COX4 and ATP5, (H) Western blot analysis of oxidative phosphorylation system complexes in hippocampus of mice (n = 3 per group), (I) ATP levels in hippocampus of mice. Values represent the mean ± SEM. n = 8 in all groups in unspecified ones. *p < 0.05, **p < 0.002, ***p < 0.0002 vs. control groups.

To further investigate the role of NAD^+^ in mitigating the impairments caused by Per3 knockout, we administered the NAMPT activator P7C3-A20 to Per3 knockout mice. Interestingly, P7C3-A20 treatment partially alleviated depressive-like behaviors, as demonstrated by behavioral assays (Fig. 5A-C).

We then evaluated the effects of P7C3-A20 on mitochondrial function in the hippocampus of Per3 knockout mice. The results revealed that P7C3-A20 administration restored the activity of SDH but had no significant effect on the activities of citrate synthase or α-ketoglutarate dehydrogenase (Fig. 5D-F). Additionally, partial restoration of mRNA and protein expression for respiratory chain complexes I-V was observed following P7C3-A20 treatment (Fig. 5G, H).

Notably, ATP levels were significantly elevated in Per3 knockout mice after P7C3-A20 administration (Fig. 5I). These findings suggest that P7C3-A20 can partially restore mitochondrial function and bioenergetics in the hippocampus of Per3 knockout mice, contributing to improvements in depression-like behaviors.

### Transactivation by PER3-BMAL1 heterodimers from NAMPT E-boxes

In mammalian cells, the NAM salvage pathway is recognized as the principal contributor to NAD^+^ synthesis, and NAMPT is recognized as the rate-limiting enzyme in the NAD^+^ salvage pathway [24]. However, whether the PER3 binds to E-boxes of Nampt regulating its expression requires further investigation.

To investigate the regulative role of PER3 on the expression of Nampt, we screened DNA sequences of Nampt and its proximal regions that might allow direct interactions. Through bioinformatics analysis, we identified two DNA sequences of putative E-boxes within Nampt that were highly conserved in mice [25]. One of the sites was located within 454–488 bp (E-box1) of intron1, downstream of the transcriptional start site, while the other (E-box2) was located in the 902–916 bp region of intron 9. Thus, we investigated whether these sites were correlated with the Per3-mediated activation of Nampt.

**Fig. 6:**
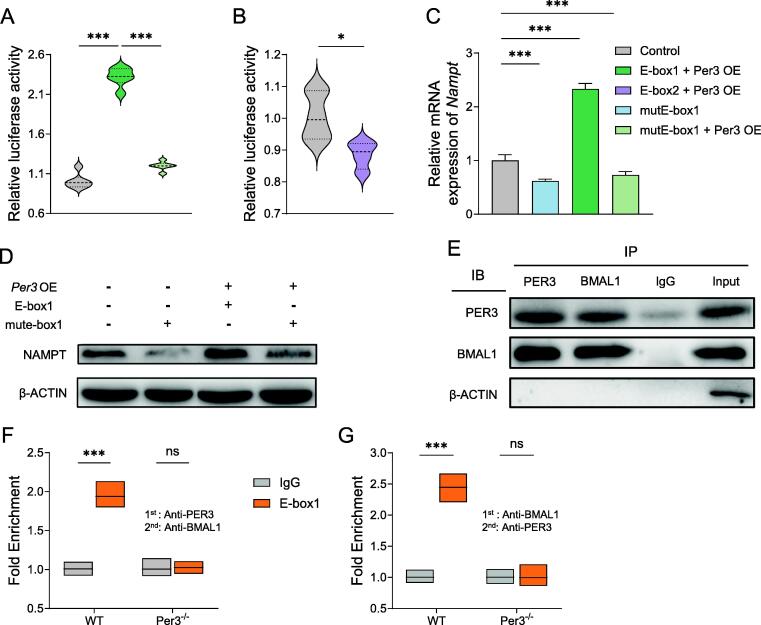
The expression of Nampt is directly modulated by PER3 and BMAL1. (A) Luciferase assay in HEK293T cells transfected with reporter plasmids containing Nampt WT E-box1 or reporter plasmid with E-box1 mutated sequence co-transfected with Per3 overexpression plasmid. (B) Luciferase assay in HEK293T cells transfected with reporter plasmids containing Nampt WT E-box2 co-transfected with Per3 overexpression plasmid. (C, D) Effects of E-box1 mutation and Per3 overexpression on Nampt mRNA and protein expression. (E) Co-IP assay was performed using anti-BMAL1, and anti-PER3 antibodies. (F, G) ChIP-re-ChIP analysis using antibodies against PER3, BMAL1, or IgG control at E-box1 site of Nampt gene in hippocampus primary cells isolated from WT and Per3 knockout mice. n = 8 in all groups in unspecified ones. Values represent the mean ± SEM. *p < 0.05, **p < 0.002, ***p < 0.0002 vs. control groups.

We, therefore, constructed two luciferase reporters containing E-box1, and E-box2 sequences, respectively. We then ectopically co-expressed Per3 and luciferase reporter in HEK293T cells. The E-box1 transfected cells showed higher luciferase activity compared to that of E-box2 cells, following the Per3 overexpression (Fig. 6A-B), suggesting that E-box1 plays a stronger role in regulating NAMPT’s transcriptional activity than its counterpart E-box2. To determine the role of E-box1 in driving NAMPT activity, we introduced a point mutation in the canonical E-box1 sequence, which abolished its enhancement of mRNA and protein expression of Nampt (Fig. 6C, D). Furthermore, we found that E-box1 mutation abolished the stimulative impact of Per3 overexpression on Nampt expression (Fig. 6C, D).

It is now well-established that PER proteins play an essential role in regulating the expression of genes by interacting with the transcriptional-translational feedback loop of clock proteins. BMAL1 is a core clock element that drives the transcription of Per and Cry genes by binding their E-box elements [26]. Therefore, we investigated whether PER3 would associate with BMAL1 to regulate the transcriptional activity of the CLOCK-BMAL1 axis in a transcriptional-translational feedback manner. To verify this, we performed a Co-IP assay, and found that immunoprecipitating PER3 led to co-precipitation of BMAL1, and in reverse, we found that immunoprecipitating BMAL1 led to co-precipitation of PER3 (Fig. 6E). Taken together, PER3 is a constituent of the endogenous BMAL1 complex.

To investigate whether PER3 and BMAL1 co-occupy the E-box1 site of the Nampt gene, we conducted a ChIP-re-ChIP assay. Initially, immunoprecipitation was performed using an anti-PER3 antibody, followed by a second immunoprecipitation with an anti-BMAL1 antibody. The results demonstrated co-occupancy of PER3 and BMAL1 at the E-box1 site in isolated primary hippocampal cells from WT mice. This co-occupancy was absent in cells from PER3 knockout mice (Fig. 6F). The reverse immunoprecipitation sequence, using anti-BMAL1 first and anti-PER3 second, produced consistent results (Fig. 6G).

These findings confirm that BMAL1 and PER3 bind together at the E-box1 element to regulate Nampt expression.

As a previous study reported that overexpression of Bmal1 can up-regulate the expression of Sirt3 and Sod2, and reduce mitochondrial dysfunction induced by 3-MCPD [27], in conjunction with our findings, we suspected that SIRT3 might also be involved in the development of depression modulated by Per3.

### Effects of Sirt3 on mitochondrial function in isolated hippocampus primary cells from Per3^−/−^ mice

**Fig. 7:**
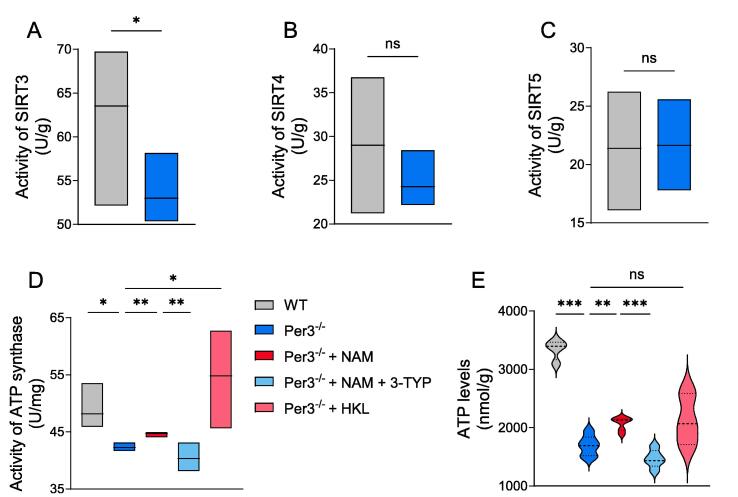
**Effects of Sirt3 on mitochondrial function in hippocampus primary cells.** (A-C) Activity level of SIRT3, SIRT4 and SIRT5, (D) Activity of ATP synthase, (E) ATP levels in hippocampus primary cells. Values represent the mean ± SEM. n = 8 in all groups. *p < 0.05, **p < 0.002, ***p < 0.0002 vs. control groups.

NAD^+^ is an important cofactor for NAD^+^-dependent enzymes of SIRTs. The mammalian SIRTs family is composed of seven members (SIRT1-SIRT7) with different subcellular localizations, and SIRT3, SIRT4, and SIRT5 are located in mitochondria [28]. Thus, we detected the activities of SIRTs that are located in mitochondria, and found the significant reduction of SIRT3 activity, but no significant change was observed in the activities of SIRT4 and SIRT5 (Fig. 7A-C), suggesting Per3 knockout may specially target SIRT3 to alter the function of mitochondria.

We found that NAM administration significantly increased ATP synthase activity in primary hippocampal cells from Per3 knockout mice (Fig. 7D). Further investigation revealed that treatment with 3-TYP, a specific inhibitor of SIRT3, reduced ATP synthase activity, while treatment with HKL, a specific activator of SIRT3, increased it (Fig. 7D). A similar trend was observed in ATP levels in primary hippocampal cells from Per3 knockout mice, although HKL administration did not result in a statistically significant change in ATP levels (Fig. 7E). Together, these findings suggest that Per3 modulates mitochondrial function through the NAD^+^-SIRT3 pathway.

## Discussion

Per3 variants are associated with altered diurnal preference and mental disorders [29]. Previous clinical studies indicated that DNA methylation of Per3 exerted a negative regulatory effect on mental health [30], and heterozygosity of Per3 allele is sufficient to exert a depressive and anxiogenic phenotype [31]. Interestingly, there is a negative correlation between gray matter volume in the right hippocampus of bipolar depressive patients and Per3 mRNA levels [32]. The polymorphism of Per3 is correlated with the depressive phenotypes in different age groups [30], [33]. These findings supported our present finding that Per3 knockout mice displayed depression-like behavior, which was consistent with a previous study [10]. However, the mechanism underlying this phenotype remains unclear. In the present study, we found Per3 regulates depression-like behaviors and these were associated with the NAD^+^-modulated mitochondrial function in the hippocampus of mice.

We used GC–MS of hippocampus tissue in WT and Per3 knockout mice, and KEGG analysis to show that the Per3 knockout was highly correlated with the notable changes in oxidative phosphorylation, oxidative stress, and energy metabolism, which are all important characterizations of mitochondrial function [34], [35]. Thus, we hypothesized that Per3 knockout-induced depression may be associated with impairment of mitochondrial function in the hippocampus. In the present study, GC–MS analysis showed that the levels of L-kynurenine and increased AFMK were significantly decreased in the hippocampus of Per3 knockout mice. L-kynurenine is a crucial intermediate in the tryptophan metabolism pathway, also known as the kynurenine pathway. Within this pathway, L-kynurenine can be further metabolized to produce various bioactive substances, including key precursors involved in the synthesis of NAD^+^ [36]. AFMK is an enzyme within the kynurenine pathway that participates in the conversion of L-kynurenine into downstream products. AFMK catalyzes the reaction that transforms L-kynurenine into kynurenic acid, representing a branch of the kynurenine pathway [37]. In our study, despite a significant decrease in L-kynurenine metabolites detected in the hippocampal tissue of Per3 knockout mice, AFMK was found to be significantly elevated, which is a seemingly contradictory result. We hypothesize that PER3 may regulate mitochondrial energy metabolism and control NAD^+^ synthesis through alternative pathways. In mammals, another crucial pathway for NAD^+^ synthesis is the NAD^+^ salvage pathway. NAMPT catalyzes the conversion of NAM into NMN, a key step in the NAD^+^ salvage pathway that allows cells to recycle NAD^+^ from NAM [38]. In this study, our data confirmed this hypothesis and showed that the decreased levels of NAD^+^, and the reduced activity of its rate-limiting enzyme of synthesis NAMPT, were observed in the Per3 knockout mice, suggesting decreased synthesis of NAD^+^ might be due to the down-regulation of NAMPT activity. As a cofactor for several enzymes, NAD^+^ is fundamental to the cellular bioenergetic metabolisms [39]. A reduction in NAD^+^ levels leads to mitochondrial dysfunction and metabolic abnormalities. Recent studies have identified that supplementation with NAD^+^ precursors, including NAMN [40], NR [41], and NAM [42], inhibiting the activity of NAD^+^-consuming enzymes can increase the NAD^+^ level and improve the overall energy metabolism [43]. These reports support our observations that NAM administration in Per3 knockout mice for 2 weeks can elevate the level of NAD^+^. Additionally, elevated NAMPT activity was detected in Per3 knockout mice, which was consistent with the previous observations that boosting NAD^+^ levels by administration of NAD^+^ precursors could activate the NAMPT [22]. In our study, supplementing with NAM ameliorated the mitochondrial dysfunction and restored the capacity of ATP generations in Per3 knockout mice.

Previous studies have showed that there are canonical or non-canonical E-box motifs in the promoter and first intron of Nampt gene [25], [44]. In combination with bioinformatics analysis and Co-IP assay, we reveal that PER3 binds the canonical E-box in the first intron of the Nampt gene, but not the other three non-canonical E-boxes in the promoter region. In our results, transduction or knockout of Per3 in hippocampus primary cells appeared to either upregulate or downregulate the Nampt expression, suggesting that Per3 might directly regulate NAMPT-mediated NAD^+^ biosynthesis. Particularly, our data showed that PER3 and BMAL1 form a complex to execute this function, which was consistent with a previous study showing PER proteins can form a complex with other clock components [45]. Hence, these data demonstrate that PER3 can directly regulate NAMPT expression in complex with BMAL1 bound to the canonical E-box.

Our previous study showed that NMN administration alleviated CORT-induced depression-like behavior and is associated with that NMN supplementation increased NAD^+^ levels to enhance SIRT3 activity [46], [47], [48], which is similar to the findings that resveratrol rescued chronic unpredictable mild stress-induced depression-like behavior [49] and kaempferol exhibited the antidepressant effect [50] through activating SIRT3 related pathways. Therefore, SIRT3 is recognized as pharmacological targets in neuropsychiatric disorders, such as depression [51]. These findings supported our result that Per3 modulated NAD^+^ level to activate SIRT3 that help to rescue the production of ATP in Per3 knockout mice. Decreased levels of NAD^+^ often induce mitochondrial dysfunction and nuclear DNA damage, highlighting the significance of maintaining intracellular NAD^+^ levels for mitochondrial stability and cellular metabolism [52], [53]. The NAD^+^ level has a strong metabolic impact because it acts as a cofactor for SIRTs, including SIRT3, a histone deacetylase that regulates a wide range of mitochondrial proteins [54]. These studies explained our observation that Per3 regulated the depression-like behavior through control of NAD^+^ level in the hippocampus of mice. Once again, such effect of NAM-mediated mitochondrial/ATP restoration is mainly dependent on the NAMPT/NAD^+^-SIRT3 driven pathway in Per3 knockout mice [22], [55], [56].

There are some essential limitations should be considered when interpreting the present results. For instance, the impact of NAM on directly modulating the physiological functions of hippocampus, especially the alterations of mitochondrial function, to rescue the depression-like phenotype in mice remains to be further elucidated. The generation of mice with selective Per3 knockout in hippocampal neurons would be useful in this respect and the injection of NAM into the hippocampus might also be useful to assess subsequent mitochondrial function and to depressive-like behaviors providing an appropriate vehicle could be found.

In conclusion, our study reveals the specific mechanisms of Per3 ameliorates depressive disorder through modulating NAMPT-controlled NAD^+^ levels and highlight a critical role for Per3 in mitochondrial energy metabolism by impacting the BMAL1 compound through the NAMPT/NAD^+^-SIRT3 pathway. These findings suggest that Per3 is a potential therapeutic target for mitochondria-related diseases.

## Compliance with ethics requirements

All animal experiments were approved by the Animal Care and Use Committee at Zhejiang University of Technology and abided by the Guide for the Care and Use of Laboratory Animals of the National Institutes of Health.

## Funding

This work was supported by grants from 10.13039/100007219Shanghai Pujiang Program (24PJD096 to X.X. Xie), 10.13039/100007219Shanghai Mental Health Center (2024-QM05 to X.X. Xie), 10.13039/100014717National Natural Science Foundation of China (31701028 to X.X. Xie), the Natural Science Foundation of Guangdong Province (2023A1515011728 to Z.Z. Li), Plan on enhancing scientific research in GMU (02-410-230221XM to Z.Z. Li), and Guangzhou Municipal Key Discipline in Medicine (2025-2027 to Z.Z. Li). All funding had no role in study design, data analysis, paper submission and publication.

## Declaration of competing interest

The authors declare that they have no known competing financial interests or personal relationships that could have appeared to influence the work reported in this paper.
