# Supplementary figures and images for: Period3 modulates the NAD+-SIRT3 axis to alleviate depression-like behaviour by enhancing NAMPT activity in mice
Source: J Adv Res. 2025 Feb 1;77:309–20. doi: 10.1016/j.jare.2025.01.043 (PMC12627864; doi:10.1016/j.jare.2025.01.043)

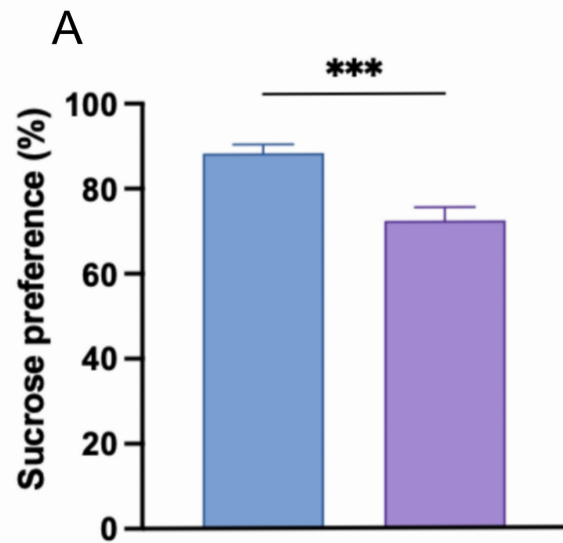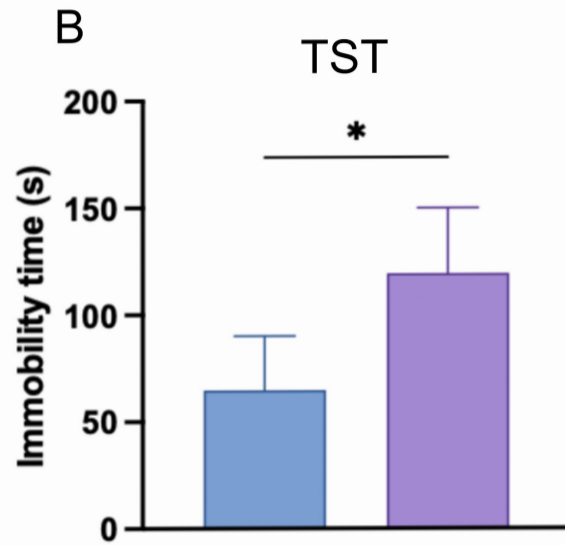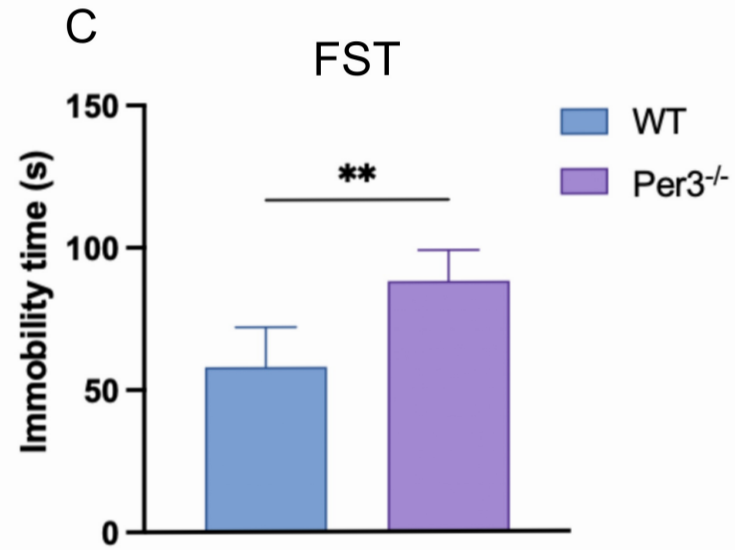

Supplement: Supplementary Data 1 [file mmc1.pdf]

A

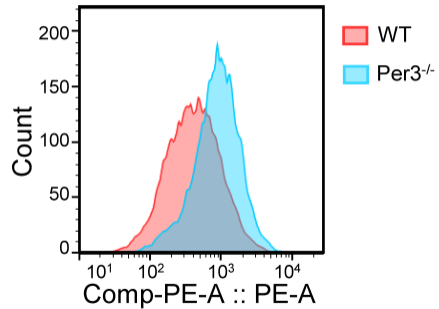

B

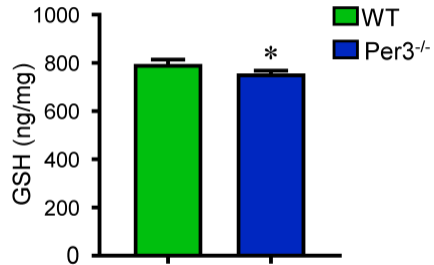

C

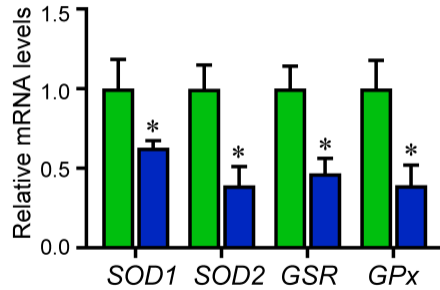

Supplement: Supplementary Data 2 [file mmc2.pdf]
